# More than a fleeting conversation: managing medication communication across transitions of care

**DOI:** 10.5694/mja2.51651

**Published:** 2022-07-31

**Authors:** Elizabeth Manias, Carmel Hughes, Robyn E Woodward‐Kron, Christine M Jorm, Guncag Ozavci, Tracey K Bucknall

**Affiliations:** ^1^ Centre for Quality and Patient Safety Research, Institute for Health Transformation Deakin University Melbourne VIC; ^2^ Queen’s University Belfast Belfast UK; ^3^ University of Melbourne Melbourne VIC; ^4^ University of Sydney Sydney NSW; ^5^ Alfred Health Centre for Quality and Patient Safety, Alfred Health Partnership, Alfred Health Melbourne VIC

**Keywords:** Continuity of patient care, Physician‐patient relations, Clinical decision‐making, Aged, Quality of health care, Health communication, Patient safety, Prescribing; Informed consent

Tailored medication communication is key to preventing patient harm across transitions of care

Older patients are likely to have complex medication regimens, which need to be carefully managed as they move across and within diverse settings, including primary care, acute care, geriatric rehabilitation, and aged care facilities. These movements often involve different health professionals, such as general practitioners and medical specialists. Problems with medication communication across transitions of care are the key reasons for increased risk of medication‐related problems and hospital readmissions.^1^, ^2^, ^3^

Discussions with older patients and families are often not prioritised across transitions of care; instead, fleeting conversations take place at irregular time points and for short periods just before or after transfers.^4^ These conversations are rarely organised in a goal‐directed way where medication communication is conveyed accurately, clearly and comprehensively. The impact of fleeting conversations is that even if medication information is conveyed, patients and families may not be involved in key decisions about newly prescribed, ceased or changed medications, or may not voice their concerns and preferences about the medication regimen.^5^ There is lack of recognition that “the one person who remains constant is the patient, who has the most to lose in a disconnected health system”.^6^

## Patients and families are excluded from conversations about managing medications across transitions of care

Communication for shared medication decision making across transitions of care is largely disorganised and serendipitous.^7^, ^8^ Shared medication decision making involves health professionals, older patients and families communicating together to define the medication problem, outlining available options, checking understanding, eliciting patient and family values, supporting patient and family deliberation, and reaching mutual agreement.^9^ Currently, older patients and families have to display extensive fortitude and perseverance to express their view.^7^, ^8^ For many health professionals, medication communication comprises information‐giving and information‐receiving activities,^10^ where they do not enquire about older patients’ experiences, beliefs and attitudes about medication .^7^, ^8^ Eliciting patient and family priorities and preferences in medication decision making is often perceived as challenging, impractical and time consuming.^7^, ^8^ Nevertheless, if medication communication is to facilitate shared decision making, a shift is needed from information‐giving and information‐receiving consultations to interactive, tailored and deliberate conversations along the continuum of care.^11^ Although not all patients and families may want to be involved, creating opportunities will help to reduce fear and anxiety and to support those unwilling or unable to participate.

Many factors contribute to health professionals’ ability to facilitate engagement with older patients and families.^5^ Older patients with complicated medication regimens, those with mental health problems, those from lower socio‐economic backgrounds or low literacy, and Aboriginal and Torres Strait Islander and migrant populations are particularly at risk.^6^ Older patients may wish to evade responsibility for fear of making inappropriate decisions, or may believe they lack the ability to participate because of language, cultural, cognitive, and disease‐related barriers.^8^

Related to medication decision making is the ethical and legal requirement to obtain informed consent before prescribing medication.^12^ If the older patient lacks decision‐making capacity, their substitute decision maker such as a family member should make the decision. They have the right to decline prescribed medication, even if this view is contrary to medical recommendations. Similarly, they may change their mind and withdraw consent about prescribed medication at any time.^12^

## A consistent practice of medication engagement with older patients and families should be our aspiration

Medication engagement should occur across transitions of care and replace fleeting conversations, using resources to assist older patients and families to communicate effectively.^13^, ^14^ The Older Persons Advocacy Network (OPAN) has a suite of materials, including video recordings in various languages, to support older patients and families in making decisions about medications. .^15^, ^16^ The following principles can help to cultivate medication communication that shifts beyond fleeting conversations.^7^, ^8^

- Communication about medication should occur throughout the duration of older patients’ care rather than limited to particular time points.
- Families should be included in medication communication at every opportunity, at ward rounds, family meetings, bedside discussions, and general practitioner discussions, rather than waiting until medication counselling occurs just before hospital discharge or just before completing a primary care consultation. Families should be informed about when these discussions are to occur so that they can plan to be available.
- Communication needs to be tailored to each patient’s ability to comprehend with clear, easy‐to‐understand language, using resources including diagrams, photographs of medications, audio and video recorded materials, simulations and patient case scenarios.
- Doctors, nurses, pharmacists and other health professionals need to acknowledge they all have important roles in communicating with each other about medication across transitions of care.
- Health professionals need to regularly seek out patient and family priorities and preferences, especially if medication changes are made. Older patients and families should be encouraged to ask questions about the medications prescribed — what medication and non‐medication options are available, what are the benefits and possible harms, what costs are involved, how are medications to be taken and for how long.^16^
- Shared decision making is supported by communicating with patients and their families about the current medications they take, the consequences that may occur if medications are not consumed, the time when these are reviewed to decide if they will be continued, and the person who conducts the review. This deliberation enables older patients and families to have a say in whether or not they agree or disagree with these decisions. Their understanding can be checked by asking them to reiterate in their own words what has been communicated to them.
- In facilitating informed consent to prescribing medications, decision aids can be helpful. Their use should be documented in medical records for future retrieval.

## Conclusion

Fostering engagement among older patients and families and creating opportunities for decision making about medications are crucial for improved safety and quality across transitions of care. Challenging fleeting conversations is key to reducing the risk of medication‐related problems and patient harm.

## Open access

Open access publishing facilitated by Deakin University, as part of the Wiley ‐ Deakin University agreement via the Council of Australian University Librarians.

## Competing interests

No relevant disclosures.

## Provenance

Not commissioned; externally peer reviewed.
